# Efficacy of Danshen injection combined with Calcitriol and Calcium/Vitamin-D for the treatment of osteoporotic fractures: A retrospective case-control study

**DOI:** 10.12669/pjms.40.3.8513

**Published:** 2024

**Authors:** Huihui Li, Dinghua Lin, Hongxin Lin, Min Liu, Guangqian Lu

**Affiliations:** 1 Huihui Li, Department of Orthopedics and Traumatology of TCM, The Third Affiliated Hospital of Wenzhou Medical University, Wenzhou, Zhejiang Province 325200, P.R. China; 2 Dinghua Lin Department of Orthopedics and Traumatology of TCM, Rui An Hospital of Traditional Chinese Medicine, Wenzhou, Zhejiang Province 325200, P.R. China; 3 Hongxin Lin, Department of Orthopedics and Traumatology of TCM The Third Affiliated Hospital of Wenzhou Medical University, Wenzhou, Zhejiang Province 325200, P.R. China; 4 Min Liu, Department of Joint surgery, The Third Affiliated Hospital of Wenzhou Medical University, Wenzhou, Zhejiang Province 325200, P.R. China; 5 Guangqian Lu, Department of Orthopedics and Traumatology of TCM, The Third Affiliated Hospital of Wenzhou Medical University, Wenzhou, Zhejiang Province 325200, P.R. China

**Keywords:** Danshen injection, Calcitriol, Calcium/Vitamin-D, Osteoporotic fractures

## Abstract

**Objective:**

To explore the efficacy of Danshen injection combined with calcitriol and calcium/Vitamin-D in the treatment of osteoporotic fractures.

**Methods:**

This was a case–control study. We retrospectively reviewed clinical data of 91 patients with osteoporotic fractures who received treatment in Rui’an People’s Hospital from February 2021 to July 2022. The data were divided into a control group with 44 records of patients who received treatment with calcitriol and calcium/Vitamin-D, and a study group with 47 patients who received Danshen injection combined with calcitriol and calcium/Vitamin-D. The control group individuals were coordinated with the patients in terms of their age and gender. Treatment effects, inflammatory response levels, and bone metabolic status levels were comparable between the two groups before and after the treatment.

**Results:**

The total efficacy of the treatment in the study group was better than that in the control group (*P*<0.05). After the treatment, levels of serum inflammatory factors in both groups decreased compared to those before the treatment, and the study group displayed lower levels than the control group (*P*<0.05). After the treatment, the bone metabolism status of both groups improved, and the improvement effect of the study group was better (*P*<0.05). The incidences of adverse reactions were similar in both groups (*P*>0.05).

**Conclusions:**

Danshen injection combined with calcitriol and calcium/Vitamin-D for the treatment of osteoporotic fractures can effectively reduce inflammation, regulate bone metabolism, and improve fracture treatment efficacy with a favorable safety profile.

## INTRODUCTION

Osteoporosis is a multifactorial metabolic bone disease characterized by a reduction in bone tissue volume per unit.^1^,^2^ It commonly affects middle-aged and elderly populations.^1^ Its most prevalent complication is osteoporotic fracture with an incidence that can reach 15%^2^ and is increasing with the extension of life expectancy, posing a serious threat to the quality of life, physical and mental health of patients.^1^-^3^

Surgical approaches are important for the clinical treatment of osteoporotic fractures.^4^ However, conservative treatment may be a better option for patients with mild symptoms and signs or those who cannot tolerate surgery.^5^ Calcitriol and calcium/Vitamin-D are commonly used for supportive treatment while other means of conservative treatment for fractures is being done.^6^,^7^ Calcitriol is a 1,25-dihydroxymetabolite that promotes intestinal calcium absorption and stimulates osteoblast activity.^6^ Calcium carbonate in combination with Vitamin-D is a bone anabolism supplement.^7^ Danshen, also known as Salvia miltiorrhiza Bunge, is a traditional Chinese herb that has been used to treat osteoporosis and has a high safety profile.^8^ Danshensu and Salvianolic acid B have been identified as the main active ingredients of Salvia miltiorrhiza Bunge aqueous extract.^9^ They have shown to promote blood circulation, unblock collaterals, remove blood stasis, and relieve pain.^10^

A recent study has shown that Danshen combined with liquefied calcium supplement has an inhibitory activity in ovariectomized mice.^11^ However, the efficacy of Danshen injection in combination with calcitriol, calcium, and Vitamin-D for treating osteoporotic fractures, as well as its impact on patient inflammatory response and bone metabolism, remain unclear. Therefore, the aim of this study was to analyze the clinical data of patients with osteoporotic fractures who received Danshen injection combined with calcitriol and calcium/Vitamin-D in our hospital to assess the clinical efficacy of this treatment plan.

## METHODS

This was a case–control study. We retrospectively selected the records of 91 patients with osteoporotic fractures who had conservative treatment in Rui’an People’s Hospital from February 2021 to July 2022. Our cohort included 40 men and 51 women; 44 patients received calcitriol and calcium/Vitamin-D (control group), and 47 received Danshen injection combined with calcitriol and calcium/Vitamin-D (study group). The control group individuals were coordinated with the patients in terms of their age and gender.

### Inclusion criteria:

- Patients with fractures meeting the criteria for osteoporotic fractures^12^
- Patients with diagnoses confirmed through dual-energy X-ray absorptiometry (DEXA)
- Patients older than 60 years
- Patients with complete clinical data

### Exclusion criteria:

- Patients with pathological fractures
- Patients with open fractures
- Patients with bone metabolism disorders
- Patients with secondary osteoporosis (such as osteoporosis due to multiple myeloma, rheumatoid arthritis, or hyperthyroidism).
- Patients with contraindications to medications used in the study.

### Ethical Approval

The ethics committee of Rui’an People’s Hospital approved this study with No. YJ2023035 on May 25 2023.

The patients in the control group received calcitriol and calcium/Vitamin-D. Each tablet of calcium/Vitamin-D contained 1.5g of calcium carbonate and Vitamin-D3 125 IU (Wyeth Pharmaceutical, 30 tablets/bottle, H10950029), and patients were prescribed one tablet orally every night. Calcitriol Soft capsules (Zhengda Pharmaceutical, specification: 0.25 μg/tablet, ten capsules, H20030491) were prescribed to be taken orally after lunch, two tablets at a time, once a day. The patients in the study group received Danshen injection combined with calcitriol and calcium/Vitamin-D. Calcitriol and calcium/Vitamin-D were prescribed the same as in the control group. Based on this, intravenous Danshen injection (Changshu Leiyunshang Pharmaceutical Co., Ltd.), 10-20 milliliters at a time (diluted with 100-500 milliliters of 5% glucose injection) were given to the patients in the study group.

The patients’ baseline data and relevant indicators collected before and six months after the treatment included:

### Treatment effect

**Fig.1:**
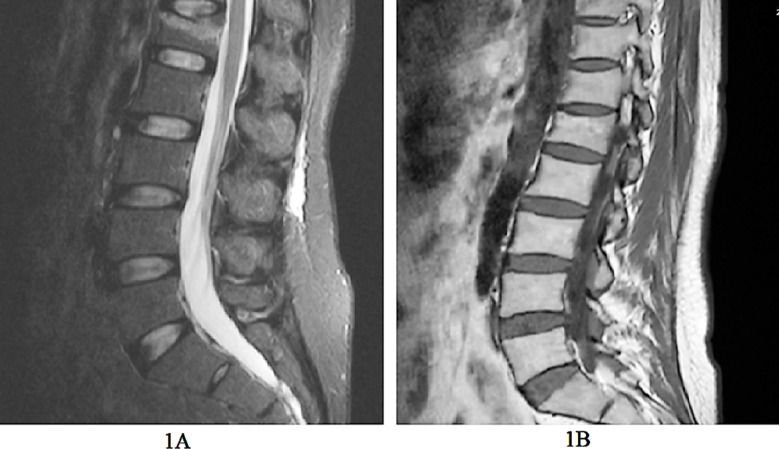
Representative magnetic resonance images of the lumbar spine of a patient with an osteoporotic fracture before and after treatment. 1A- MRI before treatment showing deformation of the first lumbar vertebral body wedge and patchy bone marrow edema; 1B- MRI after treatment showing a slightly flattened first lumbar vertebral body without bone marrow edema.

X-ray examination results after treatment was classified into three categories: significant effect, the fracture line had disappeared and the patient could engage in moderate intensity physical labor; effective, the fracture line had mostly disappeared and the patient could engage in light physical labor; and invalid, the fracture line was still present and the patient was not able to engage in physical labor. We calculated total effectiveness by taking significant effect and effective into consideration (Fig.1).

Levels of inflammatory factors: levels of tumor necrosis factor-α (TNF-α), C-reactive protein (CRP), and interleukin-6 (IL-6) were measured by ELISA from centrifuged (3000 r/minute, 15 minutes) samples of 6-mL fasting venous blood.

Bone metabolic status: C-terminal telopeptide of type I collagen (CTX-I), osteocalcin (*BGP*), and bone specific alkaline phosphatase (*BALP*) levels were measured by ELISA. The reagent kits for ELISA tests were purchased from Shanghai Mlbio Biotechnology and were used according to the manufacturer’s instructions. Records of adverse reactions.

### Statistical analysis

We conducted all data analyses using SPSS26.0 software. The normality of the measurement data was tested using Shapiro-Wilk test. Normally distributed data were expressed as means ± standard deviations and analyzed using *t*-tests. Non-normal distribution data were expressed as medians and interquartile intervals, and were analyzed using Wilcoxon tests. We presented counting data as numbers of cases and used chi-square tests or Fisher’s exact probability method to analyze them. We considered all *P*<0.05 as indicative of statistical significance.

## RESULTS

We analyzed data from 91 patients (40 men and 51 women) aged between 63 and 85 years (average, 73.20±5.48 years) with osteoporosis fracture durations ranging from two to ten months, (average, 6.27±1.81 months). Of them, 44 patients received treatment with calcitriol and calcium/Vitamin-D, while the remaining 47 received treatment with a combination of Danshen injection, calcitriol and calcium/Vitamin-D. There were 21 men and 26 women in the study group, aged between 63 and 84 years (average, 72.70±5.17 years). The osteoporosis fracture durations in the group ranged between 3 and 10 months (average, 6.49±1.79 months), and the body mass indexes between 18.9 and 28.3 kg/m^2^ (average, 23.77±2.59 kg/m^2^). There were 19 men and 25 women in the control group, aged between 63 and 85 years (average, 73.73±5.80 years), with osteoporosis fracture durations between two and nine months (average, 6.07±1.83 months), and body mass indexes between 17.9 and 28.3 kg/m^2^ (average, 23.25±2.85 kg/m^2^). We found similar baseline data variables in both groups (*P*>0.05).

The total efficacy of the study group (93.62%) was higher than that of the control group (79.55%; *P*<0.05) (Table-I). Before the treatment, the levels of inflammatory factors were similar in the two groups (*P*>0.05). After the treatment, serum levels of TNF-α, CRP, and IL-6 in both groups decreased compared to those before treatment, and the study group had lower levels than the control group (*P*<0.05) (Table-II).

**Table-I:** Comparison of clinical efficacy between the groups.

Group	n	Significant effect	Effective	Invalid	Total effective rate
Study group	47	19 (40.43)	25 (53.19)	3 (6.38)	44 (93.62)
Control group	44	17 (38.64)	18 (40.91)	9 (20.45)	35 (79.55)
*χ^2^*					3.931
*P*					0.047

**Table-II:** Comparison of inflammatory factor levels between the groups.

Time	Group	n	TNF-α (ng/L)	CRP (mg/L)	IL-6 (ng/L)
Before treatment	Study group	47	96.40±7.23	13.68±1.96	30.79±4.63
	Control group	44	97.84±7.70	14.23±2.00	31.57±4.73
	t		-0.918	-1.317	-0.796
	P		0.361	0.191	0.429
After treatment	Study group	47	49.68±5.78^a^	4.51±1.40^a^	11.21±2.96^a^
	Control group	44	67.93±6.39^a^	6.82±1.62^a^	16.98±3.63^a^
	t		-14.308	-7.296	-8.329
	P		<0.001	<0.001	<0.001

***Note:*** Compared with levels before treatment in the same group,

a P<0.05.

We found similar bone metabolism levels in the two groups before the treatment (*P*>0.05). After the treatment, serum CTX-I levels in both groups decreased compared to levels before the treatment, while the levels of BGP and BALP increased compared to those before the treatment. Moreover, CTX-I levels in the study group were lower than those in the control group, while BGP and BALP levels were higher (*P*<0.05) (Table-III). There was no significant difference in the incidence of adverse reactions between the two groups (*P*>0.05) (Table-IV).

**Table-III:** Comparison of levels of bone metabolism variables between the groups.

Time	Group	n	CTX-I (pg/L)	BGP (ng/ml)	BALP (ng/ml)
Before treatment	Study group	47	0.96±0.19	6.34±1.18	8.15±1.20
	Control group	44	0.99±0.20	6.02±1.11	8.48±1.45
	t		-0.604	1.318	-1.179
	P		0.547	0.191	0.245
After treatment	Study group	47	0.54±0.16	16.44±2.07	16.40±1.80
	Control group	44	0.68±0.19	13.52±1.84	12.57±1.61
	t		-3.742	7.105	10.697
	P		<0.001	<0.001	<0.001

***Note:*** Compared with levels before treatment in the same group, ^a^P<0.05.

**Table-IV:** Comparison of adverse reactions between the groups.

Group	n	Gastrointestinal dysfunction	Vomiting and nausea	Dizziness and headache	Total occurrence rate
Study group	47	1 (2.13)	2 (4.26)	1 (2.13)	4 (8.51)
Control group	44	1 (2.27)	1 (2.27)	0 (0.00)	2 (4.55)
*χ^2^*					0.115
*P*					0.735

## DISCUSSION

The results of this study showed that the total efficacy of the combined Danshen injection/calcitriol and calcium/Vitamin-D treatment was higher than that of calcitriol and calcium/Vitamin-D alone, while the adverse reaction rates were similar in both groups.

The efficacy of calcitriol and calcium/Vitamin-D (caltrate) in the treatment of osteoporosis has been confirmed by many studies.^6^,^7^,^13^ Calcitriol is the most active drug among the metabolites of active Vitamin-D. In a Chinese randomized control study (RCT), Zhang ZL et al^14^ showed that calcitriol can improve bone metabolism factor levels in postmenopausal women. In a review by Awadh AA et al^15^ showed that Vitamin-D supplementation can improve mobility, gait, and balance problems in elderly population (over 70 years old). A RCT by Bruyère O et al^16^ showed that supplementing calcium carbonate and Vitamin-D simultaneously resulted in better treatment compliance.

According to the traditional Chinese medicine theory, osteoporosis represents bone obstruction, withering, bone flaccidity,^17^ and it is due to spleen and kidney deficiencies; blood stasis can exacerbate damage to the spleen and kidney, leading to osteoporosis exacerbation.^18^ Traditional Chinese medicine shows that the kidney governs the bone and generates the marrow; in this context, promoting blood circulation and meridians, tonifying the kidney, and strengthening the spleen are important.^17^,^18^ Danshen injections are mainly prepared from the traditional Chinese medicine Danshen, which has a bitter taste and acts on the liver and heart meridians.^9^ Danshen is believed to ‘cool the blood’, reduce swelling, ‘clear the heart’, ‘eliminate troubles’, relieve pain, promote blood circulation, and alleviate stasis.^9^,^10^ It is also supposed to promote vascular dilation, increase blood flow, regulate local microcirculation at the fracture end, and accelerate the process of fracture healing.^9^,^10^,^19^ Wu Y et al^20^ found that Danshen administration can accelerate angiogenesis and bone regeneration in a rabbit model of ischemic necrosis of the femoral head based on mesenchymal stem cells. These results provided new ideas and a reference basis for the clinical treatment of ischemic necrosis of the femoral head. The above studies have found therapeutic value in the use of calcitriol, calcium/Vitamin-D, and Danshen injections in orthopedic diseases. However, we found no reports on the specific value of the combination of the three drugs for osteoporosis fractures.^14^-^16^,^20^ Our results indicate that the three therapies combined have high application value and are consistent with the results of the previous studies. The combined therapy seems to effectively improve the treatment effect of osteoporotic fractures and has a safe profile.

Inflammatory reactions can also have an impact on the progression and healing of osteoporotic fractures. Chen JF et al^21^ found that inflammatory factors increase dramatically during trauma and cause varying degrees of damage to the vascular endothelial function, leading to hypercoagulability and affecting the dynamic balance between bone formation and bone resorption, slowing down fracture healing and functional recovery. Ye T et al^22^ indicate that Danshen can effectively inhibit the expression of inflammatory factors, control the inflammatory response, and alleviate its adverse effects to promote recovery. Wimalawansa et al^23^ also showed that Vitamin-D is beneficial for the musculoskeletal system, improving function, enhancing immunity, and inhibiting inflammation. Our results indicated that the combination of Danshen injection, calcitriol, and calcium/Vitamin-D can effectively downregulate the expression of inflammatory factors and improve the inflammatory response. The Danshen injection has antioxidant and anti-inflammatory effects, which can clear oxygen free radicals, regulate blood hypercoagulability, and improve blood microcirculation;^9^,^10^,^22^ while calcitriol and calcium/Vitamin-D can promote improved chondrocyte metabolism and have analgesic and anti-inflammatory effects, modulating the inflammatory response.^14^,^15^,^23^

In addition, our results showed that the improvement of bone metabolism marker levels in the study group was better than that in the control group after the treatment, which suggests that the combination of Danshen injection, calcitriol, and calcium/Vitamin-D can effectively improve osteoporosis fractures of patients, aiding in their functional recovery.^24^,^25^ Both calcitriol and calcium/Vitamin-D can directly increase patient’s levels of calcium and Vitamin-D. Danshen injection has been reported to promote osteoblast proliferation, regulate bone calcium release, increase bone mass, improve bone metabolism, shorten the fracture recovery process, and enhance disease treatment effectiveness.^6^,^7^,^9^,^10^,^20^

### Limitations

This is a single-center retrospective study with a small sample size and unavoidable selection bias; the follow-up times were short, and the observation indicators few. Furthermore, baseline or after treatment VitD3 level may be a confounding variable, but they were not measured in the study. Therefore, further studies are needed to confirm specific efficacy of Danshen injection combined with Calcitriol and Calcium/Vitamin-D for the treatment of osteoporosis fractures.

## CONCLUSION

Danshen injection combined with calcitriol and calcium/Vitamin-D in the treatment of osteoporotic fractures can effectively reduce inflammation, regulate bone metabolism, and improve fracture treatment efficacy with a favorable safety profile.

### Authors’ contributions:

**HL** and **DL:** Conceived and designed the study, were involved in the writing of the manuscript and are responsible for the integrity of the study.

**HLin**, **ML** and **GL:** Collected the data and performed the analysis.

All authors have read and approved the final manuscript.
